# Editorial: Fiber-based biomaterials for tissue engineering and regenerative medicine

**DOI:** 10.3389/fbioe.2026.1839955

**Published:** 2026-05-22

**Authors:** Samaneh Ghazanfari, Pasi Kallio, Mohsen Akbari

**Affiliations:** 1 Faculty of Science and Engineering, Maastricht University, Maastricht, Netherlands; 2 Faculty of Medicine and Health Technology, Tampere University, Tampere, Finland; 3 Department of Mechanical Engineering, University of Victoria, Victoria, BC, Canada

**Keywords:** biomimetic scaffolds, cell-material interaction, controlled release systems, fiber-based biomaterials, multifunctional biomaterials, regenerative medicine, tissue engineering, wound healing

Tissue engineering and regenerative medicine increasingly rely on advanced biomaterials capable of providing structural support while dynamically regulating cellular behavior and tissue remodeling. Among the wide range of platforms explored to date, fiber-based biomaterials have emerged as particularly powerful tools due to their high surface area, tunable mechanical properties, and unique ability to recapitulate key features of the native extracellular matrix (ECM). Advances in fabrication, functionalization, and integration with biological cues have opened new opportunities for designing scaffolds that promote tissue regeneration across diverse applications. This Research Topic was conceived to highlight recent progress in fiber-based biomaterials for tissue engineering and regenerative medicine, spanning fabrication strategies, material design, biological interactions, and translational perspectives. The six contributions collected in this Research Topic reflect the interdisciplinary nature of the field and demonstrate how fiber-based and biomimetic systems can be engineered to address complex regenerative challenges in skin, cardiac, vascular, and bone tissues.

A prominent theme across several contributions is the development of multifunctional fibrous scaffolds for wound healing, where structural design is combined with bioactive and antimicrobial functions. Two original research articles present bilayer fiber-based dressings constructed from poly (lactic-co-glycolic acid)/wool keratin (PLGA/WK) and chitosan, and thermoplastic polyurethane (TPU) and chitosan, respectively. By integrating hydrophobic protective layers with hydrophilic, bioactive layers, these systems achieve simultaneous moisture regulation, infection control, and biological stimulation (Wang et al.; Wang et al.). The incorporation of agents such as nanosilver, basic fibroblast growth factor (bFGF), or zinc oxide nanoparticles enables sustained antimicrobial activity, enhanced angiogenesis, and accelerated cell migration and proliferation. Notably, the *in vivo* studies reported in these works demonstrate significantly improved wound closure and tissue regeneration compared to controls, underscoring the importance of coupling fiber architecture with controlled release of therapeutic cues. Beyond material composition, these studies illustrate how fiber morphology, layer configuration, and surface chemistry collectively regulate cell–material interactions. The reported bilayer designs exemplify how spatial control over wettability and bioactivity can be leveraged to guide the healing process, providing a framework for next-generation dressings with enhanced clinical potential.

Complementing these applied studies, another contribution explores the fundamental cell–matrix signaling mechanisms that underpin tissue regeneration (Wei et al.). Using a composite RGDmix peptide hydrogel, this work demonstrates how integrin-mediated signaling regulates growth factor secretion by human amniotic mesenchymal stem cells. By elucidating the role of the RGDSP/integrin αv/PI3K/AKT axis, the study provides mechanistic insight into how biomaterial design can enhance stem cell survival, paracrine activity, and therapeutic efficacy. This work offers a foundational design principle directly applicable to the functionalization of fibrous scaffolds, and shows how bioinspired material cues can guide tissue regeneration.

The importance of fiber architecture in guiding tissue-specific function is further highlighted in a comprehensive review focused on electrospun nanofibers for cardiac tissue engineering (Vaičiulevičiūtė et al.). This article synthesizes current knowledge on how parameters such as fiber diameter, alignment, material composition, and pore architecture influence cardiomyocyte maturation and function. Particular attention is given to emerging conductive and hybrid materials that enhance electrical coupling and functional synchronization of stem cell-derived cardiomyocytes. While significant progress has been made, the review also identifies persistent challenges, including incomplete maturation of cardiomyocytes and limitations in calcium handling and metabolic profiles. These insights provide a critical framework for advancing electrospun scaffolds toward more physiologically relevant cardiac models and eventual regenerative therapies.

Looking beyond scaffold-based regeneration, one contribution provides a bibliometric analysis of the rapidly growing field of vascular chips (Yang et al.). By systematically mapping publication trends, key contributors, and research hotspots over the past decade, this study highlights the increasing focus on endothelial biology and angiogenesis within microphysiological systems. Although distinct from traditional fiber scaffolds, vascular chips represent a complementary platform for studying cell-material interactions, vascularization, and tissue-level responses under controlled conditions. The integration of fiber-based biomaterials with organ-on-chip technologies may represent an important future direction for both fundamental research and translational testing.

Finally, the translational potential of advanced biomaterials is exemplified by a study addressing infected critical bone defects, a major unmet need in orthopedic surgery. This work evaluates a biomorphic calcium phosphate scaffold capable of local antibiotic delivery while supporting mesenchymal stem cell viability and differentiation (Salamanna et al.). The ability of the scaffold to inhibit bacterial growth and biofilm formation, while maintaining osteogenic potential, highlights how biomaterial-based drug delivery systems can simultaneously address infection control and tissue regeneration. This contribution reinforces a central message of the Research Topic: successful regenerative strategies must integrate structural support, biological compatibility, and therapeutic functionality.

Taken together, the contributions in this Research Topic demonstrate the versatility and impact of fiber-based and biomimetic biomaterials across multiple tissue engineering applications. Common themes include the importance of hierarchical structure, multifunctionality, and biologically informed design in achieving meaningful regenerative outcomes. At the same time, the studies highlight remaining challenges, such as achieving full tissue maturation, ensuring long-term functionality, and translating complex biomaterial systems into clinical practice. Looking forward, continued progress in fiber fabrication techniques, surface modification strategies, and integration with advanced *in vitro* models will be essential for bridging the gap between laboratory innovation and clinical implementation. We hope that this Research Topic will inspire further interdisciplinary research and accelerate the development of fiber-based biomaterials that can meet real-world regenerative medicine needs.

